# Optimized Surgical Positioning Using an L-Shaped Support for Arthroscopic Repair of Lateral Meniscus Tears

**DOI:** 10.1016/j.eats.2025.103944

**Published:** 2025-11-04

**Authors:** Jose Leonardo Rocha de Faria, Camilo Partezani Helito, Charlie H. Brown

**Affiliations:** a National Institute of Traumatology and Orthopedics of Brazil, Rio de Janeiro – RJ, Brazil; b School of Medicine from University of São Paulo, USP– SP, Brazil; c International Knee & Joint Center, Abu Dhabi, United Arab Emirates

## Abstract

Traumatic lateral meniscal injuries are associated with an increased risk of knee joint degeneration and osteoarthritis. Meniscal preservation through surgical repair is crucial for maintaining knee joint biomechanics. This is especially true for the lateral meniscus, given its unique anatomical and biomechanical properties. Visualization of the lateral compartment during arthroscopy is critical to access the lateral meniscus tear pattern and to determine its suitability for repair. Distraction of the lateral compartment during surgical repair of the lateral meniscus is also important to avoid iatrogenic injury to the articular cartilage. This Technical Note describes a simple surgical positioning technique to improve visualization and opening of the lateral compartment using an L-shaped support commonly used in total knee arthroplasties. The proposed method significantly improves exposure of the lateral femorotibial compartment, reducing the need for manual assistance and facilitating surgical repair, particularly in bucket-handle tears of the lateral meniscus. In addition, we discuss biomechanical considerations, surgical outcomes, and techniques to optimize lateral meniscus repair, emphasizing the benefits of inside-out and outside-in sutures. The described positioning technique and repair strategies potentially enhance surgical outcomes and long-term joint preservation.

Traumatic meniscal injuries, particularly involving the lateral meniscus, are associated with an increased predisposition to early knee joint degeneration.^1^ Studies indicate that lateral meniscal tears may raise the risk of developing osteoarthritis in the lateral knee compartment by up to 7-fold.^1^^,^^2^ The biomechanical importance of the lateral meniscus stems from the unique anatomy of the lateral compartment, where both the lateral femoral condyle and the lateral tibial plateau have convex surfaces. This significantly increases peak joint loading, even with partial losses of meniscal tissue.^3^

A recent study showed that resection of just 10% of the meniscus can lead to significant clinical and functional impairments, particularly in elite athletes.^4^ Consequently, meniscal-repair techniques have gained prominence in the literature, aiming to preserve meniscal integrity and maintain knee joint biomechanics. Biomechanical and clinical evidence supports that compared with meniscectomy, meniscal repair provides superior clinical and functional outcomes in the medium and long term, particularly for the lateral meniscus.^5^, ^6^, ^7^, ^8^ Furthermore, lateral meniscus repair tends to exhibit lower failure and complication rates, strengthening the indication for repair whenever feasible.^9^

However, adequate exposure of the lateral femorotibial compartment during surgery often requires manual varus positioning of the knee, with the operative leg crossed over the contralateral knee (figure-four position)—a technique widely described in the literature and arthroscopic surgery manuals.^10^ Nevertheless, this positioning requires an assistant to maintain it throughout the repair phase, posing an additional challenge, especially in the absence of an experienced surgical assistant.

In this Technical Note, we present a simple surgical positioning technique using an L-shaped support (Fig 1), commonly used in total knee arthroplasty procedures. This method allows for optimized exposure of the lateral compartment, facilitating meniscal repair. In addition, we share some surgical tips to assist in performing meniscal repairs, especially in bucket-handle tears extending from the posterior horn to the body of the lateral meniscus.

**Fig 1:**
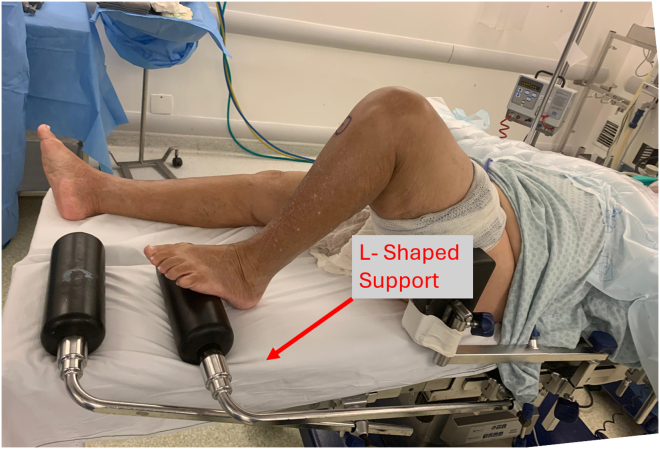
L-shaped support commonly used to maintain the knee in flexion during total knee arthroplasty procedures (left side).

## Surgical Technique

**Fig 2:**
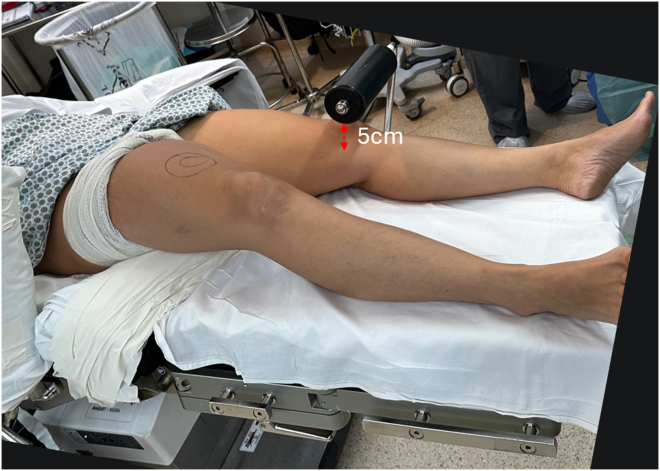
L-shaped support positioned over the contralateral knee (left side), approximately 5 to 7 cm proximal to the operative knee.

**Fig 3:**
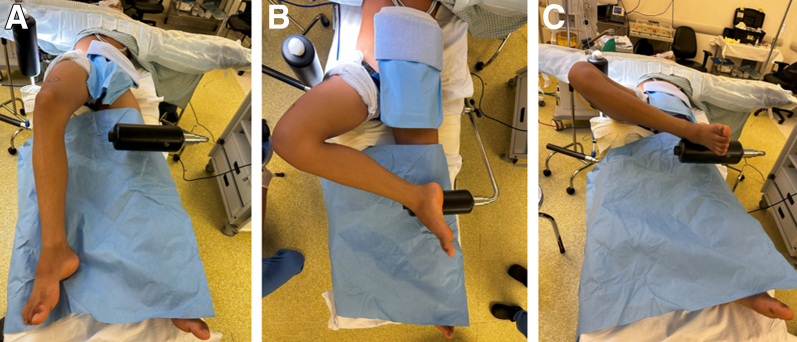
Different views of the patient properly positioned and tested before asepsis and antisepsis, in a clinical scenario in which lateral meniscus repair was already planned. (A) Frontal view of the positioning. (B) Frontal view with the operative knee flexed and the ipsilateral foot resting on the support. (C) Lateral view showing the flexed knee (right knee) with the foot securely placed on the L-shaped support.

**Fig 4:**
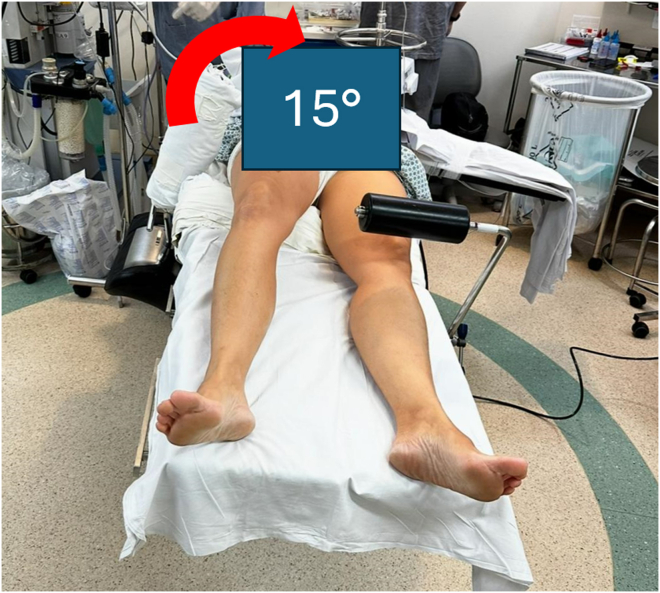
Tilt the surgical table 15° to 20° toward the nonoperative side to enhance visualization and access. This positioning facilitates the approach of both the lead surgeon and the assistant in identifying and managing the inside-out suture threads.

The patient is positioned supine on the operating table under spinal or general anesthesia. A pneumatic tourniquet is placed high on the thigh of the operative leg, and a padded lateral thigh support is positioned at the level of the anterior superior iliac spine. An padded L-shaped support, commonly used in total knee arthroplasties (Fig 1) is placed on the opposite side of the operating room table using a side rail clamp such that the support lies approximately 5 to 7 cm proximal to the patella of the contralateral knee (Fig 2). To open the lateral compartment the knee of the operative leg is flexed and the foot placed on the padded L-shaped support (Fig 3). A safety strap or belt can be placed around the patient's torso to prevent the pelvis from sliding on the surgical table. Once the patient is securely positioned on the operating room table is tilted approximately 15° to 20° toward the nonoperated side (Fig 4). Rotating the table elevates the operative leg improving access for an assistant to retrieve sutures out of a posterolateral safety incision during the repair of the lateral meniscus.

**Fig 5:**
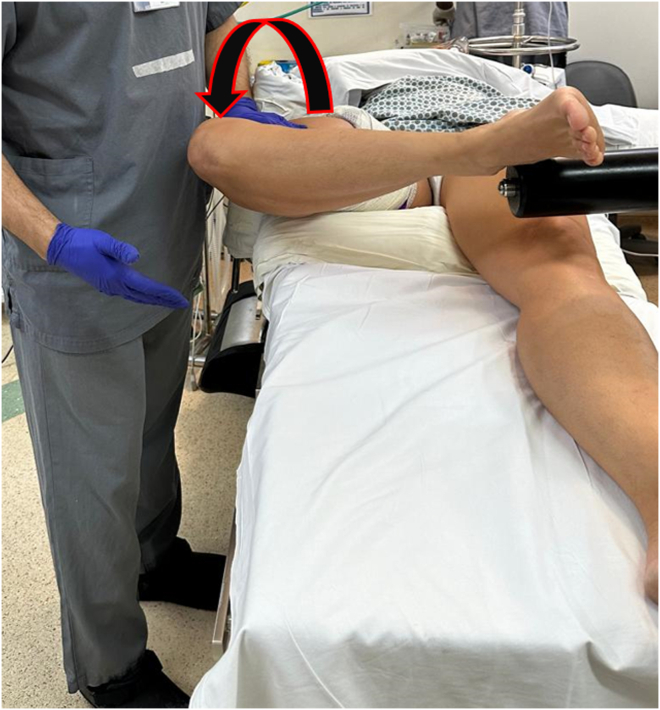
Testing the effectiveness of manual varus to open the lateral compartment before asepsis and antisepsis. Gentle pressure on the medial side of the right knee provides excellent lateral opening.

The lateral compartment is distracted and opened by placing the knee in the figure-four position. Additional opening of the lateral compartment can be obtained by the surgical assistant applying pressure on the thigh, resulting in a varus moment being applied to the knee (Fig 5). After sterile skin preparation and draping the tourniquet can be inflated and the arthroscopic portals established. Partial synovectomy is performed, allowing inspection of the patellofemoral, intercondylar, medial, and lateral femorotibial compartments.

**Fig 6:**
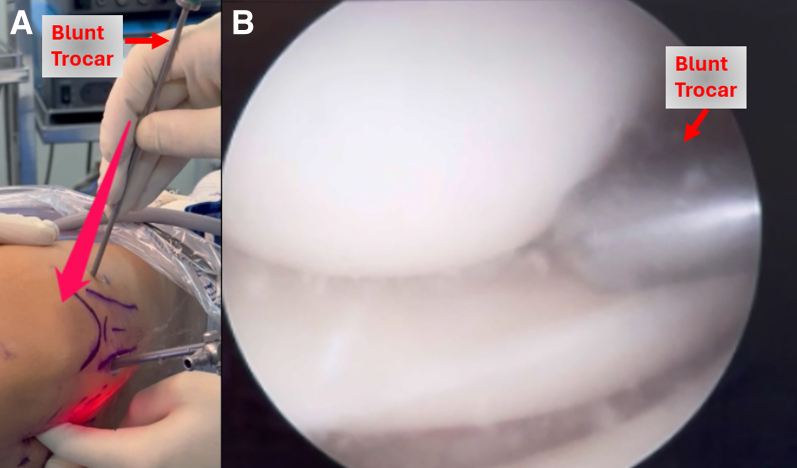
(A) View of the blunt trocar being inserted and applying pressure to the posterolateral capsule of the right knee, allowing the surgeon to palpate the path the suture device will follow. (B) Arthroscopic view of the blunt trocar approaching the joint capsule.

The assistant positions the foot on the L-shaped support while the surgeon inserts the arthroscopic camera into the lateral compartment, visualizing the displaced bucket-handle tear of the lateral meniscus (Video 1). Reduction is performed using a probe, and the lesion is assessed. For tears in the middle and posterior thirds of the lateral meniscus, a 4-cm vertical incision posterior to the lateral collateral ligament is created. The location of the posterolateral incision can be planned by pushing the tip of a blunt arthroscopic obturator against the lateral capsule at the tear site. Scissor dissection is performed between the posterior border of the iliotibial band and the superior border of the long head of the biceps tendon exposing the tendon of the lateral head of the gastrocnemius. Blunt dissection is used to open the interval between the posterolateral capsule and the tendon of the lateral head of the gastrocnemius. A popliteal retractor is placed deep to the tendon of the lateral head of the gastrocnemius to protect peroneal nerve and the popliteal vessels. Pressure from the trocar against the capsule helps determine the exit point of the inside-out suture through the incision (Fig 6).

The inside-out continuous vertical meniscal suture technique is performed: 3 sutures on the femoral surface, 3 inside-out sutures on the tibial surface of the posterior horn using the Meniscus 4-ALL device (Síntegra Surgical Sciences, Pompéia, SP - Brazil), and 2 additional all-inside sutures using the Precision device (Síntegra Surgical Sciences), near the posterior root (1 femoral, 1 tibial), totaling 8 stitches.

**Fig 7:**
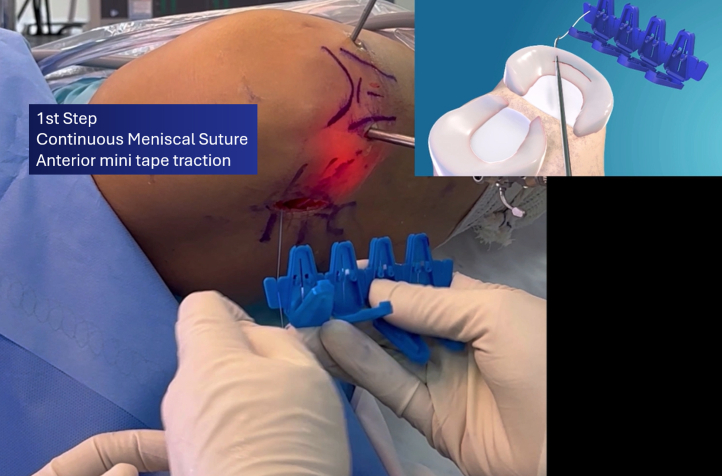
First step of the continuous vertical meniscal suture technique: anterior short minitape traction and loop fixation with the loop holder (right knee).

The continuous suture technique with the Meniscus 4-ALL consists of 3 main steps.^11^^,^^12^ First, an anterior minitape traction (short tape) is created and secured in a loop holder after traction (Fig 7). The device is then repositioned inside the joint, passed through the meniscus, and palpated externally by the surgeon’s finger on the joint capsule before completely traversing the meniscal tissue. The soft tissues are retracted, and the device is reintroduced for the second step, pulling a long posterior loop, which is then transferred to the anterior side of the device and secured again in the loop holder. This step is repeated as necessary to complete the suture; in the present case, it was repeated once, forming a second loop.

**Fig 8:**
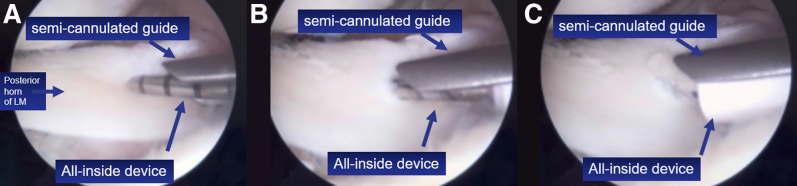
Rigid semicannulated guide used to facilitate device insertion in the desired meniscal position. (A-C) The sequence of penetration of the all-inside device (right knee).

The third and final step involves advancing the posterior loop anteriorly and cutting the anterior loop, resulting in 2 suture limbs and 2 loops. The loops are cut, and the suture limbs are tied individually. These steps are then repeated on the distal (tibial) surface of the meniscus, anchoring it posteriorly and laterally.^13^^,^^14^ The repair is completed with 2 additional all-inside sutures, one on the femoral surface and one on the tibial surface. For accurate positioning of the all-inside device and passage of the suture in the posterior horn of the lateral meniscus, a rigid semicannulated guide is used to facilitate device insertion at the desired meniscal site (Fig 8).

Use the pearls and pitfalls listed in Table 1 to facilitate your practice.

**Table 1:** Pearls and Pitfalls

Pearls	Pitfalls
Always test the positioning and stability of the flexed knee on the L-shaped support before starting asepsis and antisepsis.	Incorrect placement of the L-support can result in poor knee stabilization and compromised access.
Tilt the surgical table 15° to 20° toward the nonoperative side to enhance visualization and access.	Failing to secure the patient with a strap may lead to intraoperative shifting and surgical difficulty.
Use a safety strap or belt on the abdomen to prevent involuntary movement and sliding during the procedure.	An improper angle of table tilt can hinder visualization and limit surgical instrument maneuverability.
Use a blunt trocar to safely plan the inside-out suture pathway through tactile feedback on the capsule.	Neglecting anatomical landmarks during the posterolateral approach increases the risk of neurovascular complications.
Use a rigid, semicannulated guide to ensure precise placement of all-inside sutures in the posterior horn.	Forgetting to palpate and externally guide the device may result in misplacement of the suture device.

The rehabilitation protocol includes partial weight-bearing with the knee in extension for 4 weeks, followed by gradual functional progression. The patient is allowed to regain range of motion, limiting knee flexion to 90° during this period.

## Discussion

Our described technique of using an L-shaped support significantly improves exposure of the lateral compartment of the knee. This facilitates both visualization and surgical instrument handling, reducing the constant need for manual assistance, a particularly helpful benefit in centers with limited staff or experienced assistants (Table 2).

**Table 2:** Advantages and Disadvantages

Advantages	Disadvantages
Provides stable and reproducible positioning of the operative knee without continuous manual assistance.	Requires additional equipment (L-shaped support and side rail clamp) that may not be available in all centers.
Enhances visualization and opening of the lateral compartment, especially in bucket-handle tears.	Incorrect positioning of the support may compromise knee stability and surgical access.
Reduces the need for an experienced assistant to maintain the figure-4 position during repair.	Potential risk of patient shifting on the table if the safety strap is not properly secured.
Facilitates handling of arthroscopic instruments and suture passage in the posterolateral compartment.	Tilting the table may create ergonomic challenges for the surgical team if not well adjusted.
Increases safety during inside-out and all-inside suturing by providing consistent access and reducing iatrogenic cartilage injury.	Requires careful planning to avoid excessive varus stress or inadequate distraction of the lateral compartment.
Simple and low-cost adaptation of a device already used in total knee arthroplasty.	

The complexity of lateral meniscal repair is associated with the greater mobility of the meniscus, the more convex anatomy of the lateral compartment, and the proximity of the popliteus tendon, which limits the safe use of conventional techniques.^10^^,^^15^^,^^16^ Although several approaches have been described to increase the success rate of repair, few publications specifically address knee positioning techniques to facilitate meniscal repair procedures.^17^, ^18^, ^19^

A recent consensus recommends preserving the lateral meniscus whenever possible, even in tears previously considered irreparable, emphasizing that clinical and radiographic outcomes are significantly better compared with partial meniscectomy.^20^

Regarding the type of meniscal suture to be selected for lateral meniscus repair, a cadaveric study involving 22 knees showed that after just one flexion-extension cycle, 40% of all-inside sutures placed over the popliteus tendon failed, presenting loosening, meniscal substance tearing, and even suture breakage.^21^ Therefore, inside-out or outside-in sutures in this meniscal region may yield more promising clinical results. The practice of securing sutures with knots in the joint capsule has been shown to produce biomechanically superior outcomes.^22^

Benhenneda et al.^23^ recently reported an 86% functional success rate for repairing radial tears of the lateral meniscus in stable knees using various techniques such as inside-out and all-inside, with no significant difference observed between techniques. Using the L-shaped support facilitates the repair of radial tears in the lateral meniscus, potentially optimizing treatment outcomes for these injuries. In addition, Di Paolo et al.^24^ highlighted that repairing the lateral meniscus reduces laxity in deep knee flexion and decreases contact pressures on the tibial cartilage, contributing to long-term protection against osteoarthritis. Thus, knee positioning using the L-shaped support in conjunction with rotation of the surgical table toward the contralateral side represents a valuable strategy to facilitate lateral meniscal repair procedures.

## Disclosures

The authors declare the following financial interests/personal relationships which may be considered as potential competing interests: J.L.R.d.F. reports personal fees as a speaker from Síntegra Surgical Sciences and, reports patent holder of the Meniscus 4ALL device. C.P.H. reports personal fees as a speaker for Smith & Nephew, ConMed, Johnson & Johnson, and Med Tech. The other author C.H.B. declares that he has no known competing financial interests or personal relationships that could have appeared to influence the work reported in this paper.

## Supplementary Data

Video 1 Optimizing surgical positioning using an L-shaped support for arthroscopy repair of lateral meniscus tears. We observe a patient positioned for surgery. The L-shaped support, commonly used in total knee arthroplasty, is placed over the contralateral knee, as described in the technique. In the left image, we see a frontal view of the support positioned on the contralateral knee. In the center image, the operative knee is already flexed, and the surgical table is tilted approximately 15° toward the contralateral side, enhancing visualization of the lateral compartment and improving access for instrumentation during the procedure. Magnetic resonance imaging scans in the coronal, sagittal, and axial planes show a bucket-handle tear of the posterior horn of the lateral meniscus, with the fragment displaced anteriorly into the compartment. Before surgery begins, the knee position is tested, with the L-shaped support placed 5 to 7 cm proximal to the contralateral knee, at the level of the patella, ensuring that the knee remains stable. Gentle medial pressure is applied to confirm varus opening. Then, the surgical table is tilted 15° laterally. During arthroscopy, the lateral meniscus is seen reduced, but with an extensive tear in the posterior horn, extending into the popliteal hiatus region. A longitudinal posterolateral approach is made, parallel to the lateral collateral ligament. Scissor dissection is performed between the posterior border of the iliotibial band and the superior border of the long head of the biceps tendon, exposing the tendon of the lateral head of the gastrocnemius. Our protective landmark to avoid injury to the common fibular nerve is the biceps femoris tendon, since the common fibular nerve lies posterior to it. Therefore, by ensuring that the needles do not pass through or deep to the biceps tendon, the nerve remains protected and does not need to be directly identified. The joint capsule, the lateral head of the gastrocnemius muscle, and the popliteus tendon are then identified through blunt dissection and gentle rotational maneuvers. With the aid of a probe, the pathway of the suture device is planned and palpated externally. The transpatellar portal is used to facilitate the procedure. The inside-out continuous suture technique is then performed. The device is first introduced through the capsule, and the anterior tape suture is pulled. The device is reinserted into the joint, repositioned, and passed through the meniscus. The second step involves pulling the posterior loop and transferring it anteriorly, securing it with the loop holder. This process is repeated sequentially until the repair is complete. Sutures are also placed on the tibial surface of the meniscus, always palpating the exit point of the device before each introduction. To finish the repair, 2 additional all-inside sutures are placed—one through the tibial surface and joint capsule, and another through the femoral surface of the meniscus. A rigid semicircular cannula is used to assist with precise placement of the device, as shown in the images. The final result shows a fully repaired meniscus with inside-out and all-inside continuous sutures on both the femoral and tibial surfaces of the posterior body, with restored stability. At 7 weeks postoperatively, the patient is walking without crutches, still undergoing physical therapy, presenting good cosmetic healing with 4-cm portals and excellent progress.
